# Paper‐Embedded Roll‐to‐Roll Mass Printed Piezoelectric Transducers

**DOI:** 10.1002/adma.202006437

**Published:** 2021-01-18

**Authors:** Georg C. Schmidt, Pramul M. Panicker, Xunlin Qiu, Aravindan J. Benjamin, Ricardo A. Quintana Soler, Issac Wils, Arved C. Hübler

**Affiliations:** ^1^ Institute for Print and Media Technology Technische Universität Chemnitz Reichenhainer Str. 70 09126 Chemnitz Germany

**Keywords:** P(VDF‐TrFE), paper electronics, piezoelectricity, printed loudspeakers, R2R printing

## Abstract

The trend to a world with ubiquitous electronics has the need for novel concepts for sensors and actuators that are lightweight, flexible, low‐cost, and also sustainable. Piezoelectric transducers on the basis of functional polymers can meet these expectations. In this work, a novel concept for paper‐embedded large‐area piezoelectric devices realized solely by means of roll‐to‐roll (R2R) mass printing and post printing technologies including inline poling are introduced. The device set‐up, as well as the process technology, offers the great opportunity for a cost‐efficient and environmentally friendly mass production of thin and flexible organic large‐area piezoelectric devices. As the functional layers are embedded into paper by the hot lamination of two poly(vinylidene fluoride‐*co*‐trifluoroethylene) P(VDF‐TrFE) layers, the printed electronics is protected and invisible. The paper gives insights to the R2R printing of a 500 m long web including R2R post printing processes and electrical and acoustic inline characterization. Fully R2R processed devices show a high remnant polarization of up to 78 mC m^−2^ and can be realized with high yield of >90%. Finally, a 360° surround‐sound installation realized with a 387 cm long paper web consisting of 56 piezoelectric speakers including wiring is presented.

## Graphical abstract

Paper‐based fully roll‐to‐roll manufactured flexible lightweight large‐area piezoelectric transducers are presented. Embedding the printed electronic layers into conventional paper webs by inline lamination and inline poling leads to piezoelectric devices with high remnant polarization and high production yield. The development gives the opportunity i.a. for novel surround sound installations of several meter length.

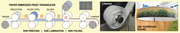

For a long time, paper has been used as storing medium for written information only. In combination with the development of printing technologies, it became one of the most relevant materials as information could be reproduced multiple times and brought to millions of people in a simple, cheap, and fast way. However, with the digital revolution the end of paper has been forecasted.

However, paper still has its big advantages. The yearly production is still huge with over 400 million tons worldwide^[^ ^1^ ^]^ for a wide application range going much beyond conventional books, newspapers, packages, or sanitary products. It is a natural light‐weight, flexible, recyclable, multi‐functional material making it an ideal candidate as part of novel electronic devices, especially based on printed electronics.^[^ ^2^ ^]^ During the last decade, a wide variety of electronic functionalities have been demonstrated with paper as the common substrate platform. It has been used as basis for organic circuits,^[^ ^3^ ^]^ microwave and digital electronics,^[^ ^4^ ^]^ sensors,^[^ ^5^, ^6^, ^7^ ^]^ actuators,^[^ ^8^, ^9^ ^]^ and many more.

Within the group of printing technologies, sheet‐fed (S2S) systems have been used most often for research and development in the field of printed electronics because of comparably simple sample handling and relatively low material consumption and overall effort. Roll‐to‐roll process technologies require a much more elaborated web handling and usually much bigger amounts of material. Thus, the available number of publications with respect to truly and fully R2R manufactured printed electronic devices is comparably small: sensors,^[^ ^10^ ^]^ solar cells,^[^ ^11^, ^12^ ^]^ organic field‐effect transistors^[^ ^13^ ^]^ were demonstrated. Often, the R2R technology is used partially for single layers of the devices only.^[^ ^14^ ^]^ This situation becomes even more obvious, when a complete inline manufacturing consisting of R2R printing and R2R post‐treatment (e.g., long annealing/sintering, poling, charging, functionalization, protecting, etc.) is required for a completion or full activation of the electronic devices.^[^ ^15^ ^]^

The same situation can be found when looking for publications dealing with printed ferro‐ and piezoelectric devices. Typically, these devices on the basis of plastic foil substrates consist of metallic or polymeric electrodes and a piezoelectric polymer (e.g., P(VDF‐TrFE)) sandwiched in between several S2S techniques have been used for the realization.^[^ ^16^, ^17^ ^]^ However, as the piezoelectric layers need sufficient drying and annealing for favorable crystallization and an additional poling procedure to achieve dipole alignment, the whole process is extremely time‐consuming and inefficient.

A further challenge for printed electronic devices is the smart and smooth integration to the environment without losing the advantages of the new kind of flexible electronics. Additionally, a “hidden electronic” is desired with the option to equip the visible surface with conventional color prints.

With our investigations we demonstrate for the first time a truly fully R2R manufacturing of novel paper‐embedded flexible large‐area piezoelectric devices including all R2R printing steps, R2R lamination, and R2R contact poling for a final roll of more than 100 meters length of so called “T‐PAPER” (“T” for “tone”). The results give insights to the quality of such a complete complex inline process creating the opportunity for cost‐efficient production of piezoelectric devices to be used for unique actuator or sensor applications. As an example, we showcase a 387 cm long continuous loudspeaker ring consisting of 56 paper‐embedded invisible speaker cells combined to a 7 segment 360° surround sound installation including fully printed wiring and color print illustration.

Technical basis for our developments were two R2R printing presses LaborMAN 1 and LaborMAN 2, both having a web width of 140 mm, used for all printing, post‐printing and inline measurements as explained in the following.

Material basis of T‐PAPER is a conventional double‐sided coated low‐cost graphic paper (maxigloss) with a thickness of 100 µm. Despite the glossy nature of the paper originally made for color prints, it has an arithmetic mean roughness *R_a_* of 394 nm, hence, much rougher than a smooth PET or PEN foil often used for printed electronics applications.

At first, large‐area electrodes (6 × 11 cm^2^ each) were made by means of rotary screen printing at LaborMAN 2 in a continuous pattern. The printing unit is an in‐house development to be compatible with the LaborMAN printing press design. As electrode material, a conductive polymer poly(3,4‐ethylenedioxythiophene) polystyrene sulfonate (PEDOT:PSS) was used as received (AGFA EL‐P 3155). For a proper ink flow and better film forming, the formulation was stirred for 2 h prior to printing. A web length of more than 500 m was printed at a printing speed of 10 cm s^−1^. The PEDOT:PSS ink was dried in two steps leading to a final layer thickness of ≈400 nm. For a very quick pre‐drying, the paper web passed a near IR (NIR) dryer directly after the printing unit. PEDOT:PSS is extremely sensitive for NIR absorption,^[^ ^18^ ^]^ thus a 2 s inline treatment is enough to evaporate most of the solvent of the ink formulation. Even the high‐boiling point solvents incorporated as a secondary dopant to the ink formulation needed for achieving high conductivity of the polymer film^[^ ^19^ ^]^ can be extracted effectively. To remove solvent residuals, additionally to the NIR drying, a conventional hot‐air dryer (140 °C) with a drying length of 8 m was used resulting in an extra drying time of 80 s at the aforementioned web speed. Afterward, the web with the electrodes was rewound.

Second, the core of the device, the piezoelectric layer, was realized by rotary screen printing too. Poly(vinylidene fluoride‐*co*‐trifluoroethylene) (P(VDF‐TrFE), 75:25 mol%, Piezotech Arkema) was employed for this layer. It can be formulated to an ink by dissolving the copolymer in a number of organic solvents such as methyl ethyl ketone, dimethyl sulfoxide, dimethylformamide, *N*‐methyl‐2‐pyrrolydone, and cyclopentanone or a mixture of solvents. These solvents are able to dissolve the co‐polymer up to a concentration of at least 25 wt% which is required for comparably thick films in the range of 5–10 µm. After printing, the web containing the wet co‐polymer layer ran at a speed of 5 cm s^−1^ through the printing press for 80 s prior the drying process, which is beneficial for a proper film forming and leveling to close pinholes typically visible directly after the printing unit. The drying process itself was done with the aforementioned hot‐air inline dryer for ≈160 s at a temperature of 150 °C. After drying, the web was rewound again.

The third printing step was used for the realization of highly conductive contact pads at one edge of the PEDOT:PSS electrodes made of silver ink (Henkel Loctite ECI 1011 E&C). Here, rotary flexo printing was utilized at a printing speed of 5 cm s^−1^. The comparably low speed is required for sufficient annealing of the silver particle ink to achieve high conductivity. The inline hot‐air drying procedure lasted ≈160 s at a temperature of 170 °C. The web, now consisting of all required layers for one half of the piezoelectric device, was rewound once more.

The complete device combines two of the above described pre‐printed paper webs. For top and bottom side, one and the same design can be used. Thus, the more than 500 m long web was split into two rolls with ≈260 m of pre‐printed material each. These rolls were fed to two separate unwinding units of our 15 m long R2R machine LaborMAN 1. Besides four printing units, the machine consists of different post‐press units including a hot lamination unit. Both webs were brought into contact in the nip of this unit with the printed P(VDF‐TrFE) top and bottom electrode surfaces face to face. According to the data sheet of the manufacturer, the used P(VDF‐TrFE) material has a melting point of 150.5 °C. Varying the web speed, nip pressure, and lamination temperature, the process was optimized w.r.t. piezoelectric and mechanical performance of the T‐PAPER laminate. The final lamination settings for this work were 3 cm s^−1^, 1 bar, 160–180 °C for the web speed, the pressure of the impression roller and the surface temperature of the lamination cylinder, respectively. It is important to notice that both in‐fed webs as well as the laminated out‐fed web have to run well‐controlled through the whole machine to achieve good alignment of the pre‐printed structures, especially the register of the bottom and top electrodes, and to avoid the formation of wrinkles.

To achieve piezoelectric devices with high remnant polarization, a poling step is needed. To do this, a fully automatic inline contact poling (and measurement) system integrated to the LaborMAN 2 was developed. It automatically detects the position of individual cells, measures the capacitance, and do the polarization in an adapted way for each and every single cell. The specific polarization procedure (following the “Bauer cyclical poling method”),^[^ ^20^ ^]^ consisting of ten sinusoidal voltage cycles is explained in more detail in the Supporting Information. As the polarization current is depending on the speed of the polarization, the web speed was set to 1 cm s^−1^ which gives maximal 6 s time for poling one single cell. For our purpose a time frame of 5 s was used to apply ten voltage cycles at a frequency of 2 Hz. The relatively slow speed in comparison to usual R2R printing speed relies on the limited power of the used high‐voltage power amplifier with a maximum output current of 80 mA and the computational power of the installed system (standard Windows PC with Matlab). In principal, the poling could be done at much higher speed as the switching of the dipoles happens in µs‐range when applying an electric field of ≈100 MV m^−1^.^[^ ^21^ ^]^ The whole manufacturing procedure, the device set‐up as well as photographs of single process steps and the final T‐PAPER roll are displayed in **Figure** **1**.

**Figure 1:**
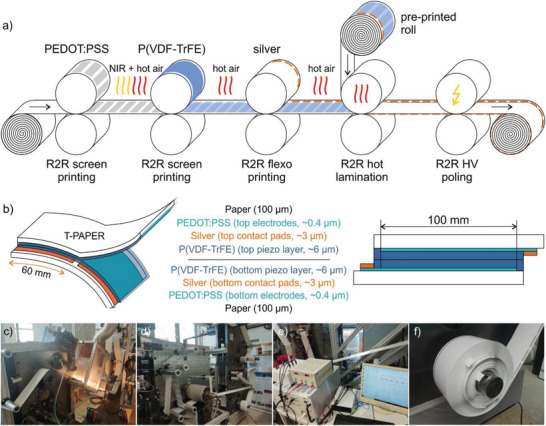
R2R manufacturing process of T‐PAPER. a) Schematic illustration of R2R printing steps, lamination and inline polarization. b) 3D schema and cross‐sectional view of the T‐PAPER device set‐up (active area per cell 60 × 100 mm^2^). c) R2R printing press LaborMAN 2, rotary screen printing of PEDOT:PSS electrodes. d) R2R hot lamination of two pre‐printed T‐PAPER rolls at LaborMAN 1 and e) HV inline polarization of laminated T‐PAPER roll at LaborMAN 2. f) Final 260 m T‐PAPER roll.

As we propose a mass production approach, it is essential to have information about the process stability from one process step to the other. Therefore, we monitored the key parameters of all layers inline. For the large‐area PEDOT:PSS electrodes, continuous inline sheet resistance measurements were conducted using a four‐point method (pin‐to‐pin distance 15 mm, **Figure** **2**a).

**Figure 2:**
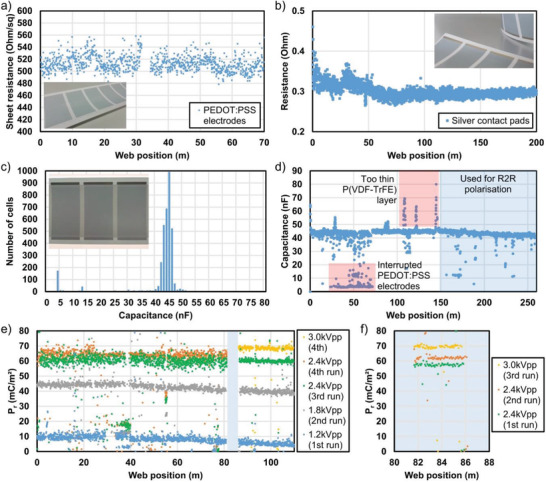
Inline measurements of different parameters: a) Inline sheet resistance measurement of rotary screen printed PEDOT:PSS electrodes. One dot represents the *R_s_* value for one single electrode. Inset: Photo of the screen printed PEDOT:PSS electrodes. b) Inline resistance 2‐point measurement of flexo printed silver contact pad lines. Inset: Photo of PEDOT:PSS electrodes with flexo printed silver contact pads. c) Inline capacitance measurements of a 260 m long fully printed and laminated T‐PAPER web: distribution of the capacitance values per cell. Inset: Photo of three laminated T‐PAPER cells. To make the printed structures visible, the photo was taken with backlight coming from a light table. d) Distribution of the capacitance values along the 260 m long laminated T‐PAPER web. e) Distribution of remnant polarization along a 110 m long inline polarized T‐PAPER web. The polarization was done stepwise in four web runs with variable polarization voltage (1.2/1.8/2.4/3.0 kV_pp_). The light‐blue rectangle in (e) (web position 82–86 m) highlights a segment used for: f) an additional inline polarization test with increased poling voltage of 2.4 kV_pp_ already for the first web run followed by a second run at 2.4 kV_pp_ and a third run with 3.0 kV_pp_.

For a 70 m long reference web, the average sheet resistance *R_s_* was 513 ± 19 Ω sq^−1^. The very low standard deviation of ±4% clearly indicates the high process stability of rotary screen printing the PEDOT:PSS formulation including the drying procedure with NIR and hot‐air dryer. The relatively high sheet resistance is caused by the nature of the natural paper substrate with its rough and porous surface which strongly influences the conductivity of layers with a thickness in the nanometer range.^[^ ^22^ ^]^ However, for low frequency applications up to a few kHz, the achieved conductivity value is still high enough to avoid large power losses.^[^ ^23^ ^]^ Furthermore, for large‐area electronics often defined as low cost electronics, it is important to find a good balance between required performance of single layers and material consumption of the functional layers which significantly influences the costs per device. Besides the economic balance, the ecologic aspect becomes high priority as well. Therefore, paper without additional plastic coating as intermediate layer was selected.

The quality of the flexo printed silver contact pads was monitored by an inline two‐point measurement method (pin‐to‐pin distance 26 mm). The method was selected because of the geometry of the pads (5 mm wide; 60 mm long) allowing for an estimation of the sheet resistance by *R_s_* = *Rwl* ^−1^, with *R_s_*, *R*, *w*, *l* being the sheet resistance, the measured resistance between the pins, the width of the contact pads, the pin‐to‐pin distance, respectively. Based on the two‐point resistance measurements on a more than 200 m long test print (Figure 2b), the mean value of the sheet resistance *R_s_* was calculated to be 56 ± 6 mΩ sq^−1^. The rather low absolute value and deviation show the high conductivity of the 3 µm thin printed silver contact pads and stability of the flexo printing process of the silver ink, respectively. Thus, the small contact pads provide homogenous voltage distribution to the polymeric large‐area electrodes from one edge with the additional benefit of a low silver consumption which is again important from economic and ecologic point of view.

A direct inline quality control of the piezoelectric P(VDF‐TrFE) layer with a total area of more than 55 m^2^ for a more than 500 m long web is challenging and can be misleading, for example, using an additional metallic roller as temporary top electrode of the printed stack with PEDOT:PSS bottom electrode and P(VDF‐TrFE) dielectric. Instead, the piezoelectric layer was characterized in two ways after completing the whole device, namely, after lamination of two identically pre‐printed and roll‐cut (to get free access to the silver contact pads, see Experimental Section) 260 m long webs each having both P(VDF‐TrFE) layers face‐to‐face. Consequently, the capacitance of more than 3500 laminated cells with 60 cm^2^ electrode overlap area each on a 260 m long roll could be inline measured (Figure 2c).

Despite the huge amount and total area of the fully printed and laminated samples, the capacitance values are quite stable with a mean value of 44.0 ± 3.1 nF (cells with *C*_meas_ < 30 nF excluded). It should be noted that the measurement includes deviations not only of the P(VDF‐TrFE) layers as dielectric but also of alignment errors in and across the web direction coming from the alignment control of the lamination of both pre‐printed webs. We estimate this error as high as max. 1 and 0.5 mm in and across the web direction, respectively, leading to an overall maximal reduction of the electrode overlap area of 2.6 cm^2^ or 4% with respect to the nominal area of 60 cm^2^. Calculating with a permittivity of 11 typical for non‐polarized P(VDF‐TrFE), the average layer thickness of the dielectric was ≈12 µm. Thus, one screen printing run resulted in a layer thickness of ≈6 µm. Additionally, the estimation of the layer thickness via the ink consumption (in total ≈4.1 kg of ink) for the whole printing run resulted in similar values. The capacitance distribution (Figure 2d) throughout all samples shows a clear Gaussian behavior with a very narrow distribution around the mean value. However, there are two exceptions visible (Figure 2c, indicated with reddish rectangles). i) There was a relatively large number of samples (5.6%) showing a very small capacitance value below 10 nF only. As a sudden increase of around four times in the layer thickness could be excluded for a continuous printing procedure, the reason was detected to be a (partial) cut/break in the PEDOT:PSS layer. Such a cut leads to an additional serial resistance to the pure capacitance (and the resistance of the electrodes themselves) in the kΩ range. The serial resistance leads to an increased frequency of the oscillator circuit used for the capacitance measurement. Hence, a lower capacitance value is calculated by the system. ii) The capacitance plot shows some narrow parts ≈1–2 m long with peak values up to 90 nF. Here, we clearly observed a reduction of the total layer thickness of the dielectric, mainly caused by a non‐fully automated ink feeding to the printing unit. In the worst case, the samples consisted only of one half of the average layer thickness, leading to a doubling of the average capacitance. It should be noted that a systematic influence of the capacitance measurement by high leakage currents was not observed. Otherwise the oscillator circuit would stop functioning as the leaky capacitor is not charging high enough. Actually, this is an interesting approach for a fast dual test (capacitance and leakage) of dielectrics with simple method.

Solution processed P(VDF‐TrFE) crystallizes directly in the polar β‐phase. However, dipoles are randomly oriented leading to a zero net polarization and the absence of piezoelectricity. Thus, poling is required for dipole alignment along an applied electric field between the electrodes. As the poling procedure requires high electric field strength of more than 50 MV m^−1^ which is a typical value for the coercive field of P(VDF‐TrFE), this process step is challenging as small inhomogeneities like pinholes in the piezoelectric layer or roughness peaks of the electrodes caused by the paper surface can lead to a local electrical breakdown damaging the whole device. Polymer electrodes demonstrated already much better polarization potential in comparison to metallic ones,^[^ ^24^ ^]^ but the polarization of paper‐embedded laminated piezoelectric devices has not been published so far.

For our studies, the last 110 m of the 260 m T‐PAPER roll showing the best stability of the capacitance values (Figure 2d, bluish rectangle) were selected for continuous inline polarization.

Around 1500 cells were polarized stepwise in four web runs to investigate the development of the polarization with a maximum poling voltage of 1.2, 1.8, 2.4, and 2.4/3.0 kV_pp_ for the first, second, third, and fourth web run, respectively (Figure 2e). The maximum voltage for the continuous inline poling was limited to ≈125 MV m^−1^ to keep the yield high. The web speed was set to 1 cm s^−1^ limited by the power of the HV amplifier. Nevertheless, even such a low web speed in comparison to a typical web speed of R2R printing presses gives the opportunity to polarize a very large number of large‐area piezoelectric devices in a short time fully automatically including full electrical characterization, in our case ≈500 devices per hour.

For samples polarized at a polarization voltage just slightly above the coercive field, a small remnant polarization of 8.0 ± 2.6 mC m^−2^ was achieved. The slightly lower polarization at the end of the web correlates very well with the small decrease of the capacitance values (max. 10% less than *C*_mean_) detected for the same web section. A single clear reason could not be identified for the decrease, thus a combination of small process deviations (printing and lamination conditions and alignment) could explain the results. In case of a slightly increased layer thickness of the P(VDF‐TrFE) the effective electric field strength for the polarization would decrease which plays an important role for low electric fields around the coercive field, but the effect becomes small for high electric fields. Increasing the polarization voltage to 1.8 kV_pp_ (≈75 MV m^−1^) and 2.4 kV_pp_ (≈100 MV m^−1^) for the second and third run already induced a polarization of 42.6 ± 2.4 and 60.3 ± 2.3 mC m^−^^2^, respectively. For the first 80 m of the fourth run we kept the poling voltage of ≈100 MV m^−1^ to see the effect of a multiple poling cycle at constant maximum electric field. Obviously, it is possible to improve the polarization with a larger number of poling cycles as the remnant polarization increased to 64.0 ± 2.1 mC m^−^^2^. Such a strategy is an effective way for a safer poling at moderate voltage while achieving high remnant polarization. For the last 30 m of the web, the poling voltage was set to 125 MV m^−1^, leading to a remnant polarization of 68.7 ± 1.4 mC m^−2^ in combination with a high cumulative yield of 93%. Additionally, Figures S3–S5, Supporting Information, show the distribution of the achieved remnant polarization for the third and fourth inline poling run. A summary of all extracted poling results is given in **Table** **1**.

**Table 1:** Summary of achieved remnant polarization values, standard deviation, and yield of a R2R manufactured 110 m long T‐PAPER web

Poling run	Poling voltage [kV_pp_]	Poled cells	*P*_r_ [mC m^–2^]	Standard deviation [mC m^–2^]	Standard deviation [%]	Cumulative yield [%]
1	1.2	≈1520	8.0	2.6	32%	97
2	1.8	≈1520	42.6	2.4	6%	92
3	2.4	≈1520	60.4	2.3	4%	91^a)^
4‐1	2.4	≈1160	64.0	2.1	3%	88^a)^
4‐2	3.0	≈430	68.7	1.4	2%	93^a)^
Static	3.6	<10	78			

^a)^ Calculated by the ratio between the number of cells with 55 ≤ *P* ^r^ ≤ 75 mC m^–2^ and the total number of poled cells.

Additionally, it should be noted: i) Higher polarization values can be achieved by applying more poling cycles at moderate poling voltage which would require more time.^[^ ^25^ ^]^ ii) Alternatively, a one‐cycle higher poling voltage can also be applied directly in order to save time. No significant decrease of the yield could be observed for a short test part of the web (Figure 2f, web position 82–86 m) starting already with 100 MV m^−1^ for the first poling run. The selected step‐wise voltage increase within one poling cycle (Figure S1, Supporting Information) seems to be beneficial to avoid destructive breakdowns. iii) Single samples were polarized at even higher electric field up to 154 MV m^−1^, leading to a maximum remnant polarization of 78 mC m^−2^ which is exceptionally high for a large‐area P(VDF‐TrFE) device on paper substrate and higher than what was recently published by our group for non‐laminated paper‐based piezoelectric devices (71.5 mC m^−2^). Hysteresis loops for representative R2R manufactured samples polarized inline or offline at different maximum electric field are shown in Figure S2, Supporting Information, for the sake of completeness. We attribute the high performance and robustness of the piezoelectric layer to the introduced lamination process step that leads to a compact pinhole‐free piezoelectric layer with low leakage and high degree of crystallization.

SEM cross sectional images and XRD measurements give a first insight into the T‐PAPER device set‐up and especially the P(VDF‐TrFE) morphology. **Figure** **3**a–c shows cross sectional SEM images of a laminated T‐PAPER sample. For a homogenous lamination of the two P(VDF‐TrFE) layers, it is essential to achieve complete melting of at least one of the layers. Consequently, no gap and air entrapment is visible between the two P(VDF‐TrFE) layers and good adhesion can be provided. If the thermal entry is too low (e.g., if the web speed is too high), only partial lamination occurs (Figure 3d). It is well known that thermal treatment affects much the crystal structure of P(VDF‐TrFE). Annealing at temperatures between the Curie (*T*_C_) and melting temperature (*T*_m_) is quite effective to improve the crystallinity and hence the ferroelectric properties. However, annealing above *T*_m_ leads to irreversible degradation of the crystallinity and consequently the ferroelectric performance.^[^ ^26^, ^27^, ^28^ ^]^ Actually, this could be confirmed by XRD measurements on T‐PAPER samples too. Figure 3e shows clear differences of the XRD diffractograms of samples laminated with different temperature and treatment time. If the temperature or the treatment duration becomes too high, the peak at ≈23° responsible for the β‐phase of the P(VDF‐TrFE) layer becomes smaller. Consequently, the achieved remnant polarization for these samples varied as well (see legend of Figure 3e). These results are in qualitative agreement with previous studies.^[^ ^28^ ^]^ In our investigation, we controlled the temperature of the lamination cylinder, and did not monitor the exact temperature of the sample. Since the cylinder temperature is well above the *T*_m_ of the P(VDF‐TrFE) films (150 °C according to the data sheet of the manufacturer), we believe that the main reason for the decrease of the crystallinity with longer treatment time is because higher temperature is reached at the P(VDF‐TrFE) films.

**Figure 3:**
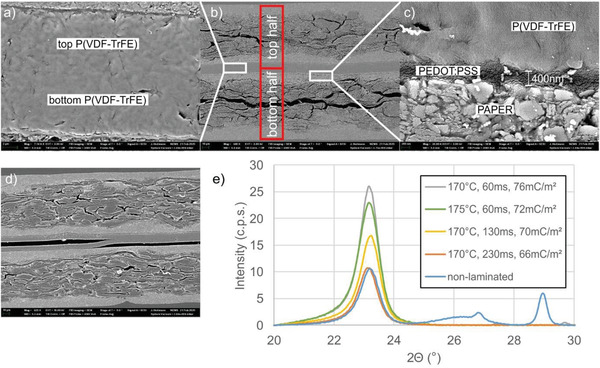
SEM cross sectional images of hot laminated T‐PAPER samples. a) Detail of two laminated P(VDF‐TrFE) layers. b) Overview image showing both T‐PAPER halves. c) Detail of the interface paper coating/PEDOT:PSS/P(VDF‐TrFE). d) Insufficiently laminated P(VDF‐TrFE) layers. e) XRD diffractograms of P(VDF‐TrFE) layers laminated with different process parameters (cylinder temperature, treatment duration).

Devices with such high remnant polarization can be used as flexible piezoelectric sensors and actuators. Here we demonstrate the functionality of the R2R produced samples as paper‐embedded audio loudspeakers.

To get an overview of the homogeneity of the sound performance along the 110 m polarized T‐PAPER web, we performed an inline audio measurement directly at the R2R printing press. To the best of our knowledge, an acoustic test of R2R printed electronic devices within a printing press has not been published before. At first, the acoustic response of a single T‐PAPER cell to a linear frequency sweep from 500 Hz to 20 kHz and a sinusoidal peak‐to‐peak voltage of *V*_pp_ = 100 V created by a signal generator in combination with an audio amplifier was recorded in static conditions, that is, without moving web. It shows a typical frequency response for piezoelectric thin film speakers with best performance in mid and high audio frequency range from ≈2 kHz to more than 10 kHz (**Figure** **4**a). It should be noticed that the frequency behavior strongly depends on the size of the speaker cells. The larger the cells, the more pronounced is the lower frequency output which can be used for large‐area sound installations. For the evaluation of the moving web, we applied a fixed sinusoidal waveform with a frequency of 4.9 kHz and a peak‐to‐peak voltage of *V*_pp_ = 100 V to the individual cells while running the web through the machine with a speed of 2 cm s^−1^. The frequency response was recorded by means of a microphone placed 5 cm above the geometrical center line of the piezoelectric cells without any noise cancellation. Such a measurement set‐up gives a very good signal‐to‐noise ratio despite the big noise of the printing press which is mainly located in the low‐frequency range. The recorded audio signal was FFT analyzed with the help of the audio software Samurai to extract the sound pressure level (SPL) of the relevant 4.9 kHz signal (1st harmonic). It should be noticed that the total harmonic distortion for the applied 4.9 kHz signal was very low (0.67%). The mean peak SPL value at 4.9 kHz was measured to be 86.1 dB while 93% of all cells (which is in very good agreement with the cumulative yield of the whole complex R2R manufacturing process—see Table 1) showed an SPL of more than 82 dB, confirming the very good stability of the process and the device set‐up including material selection (Figure 4b).

**Figure 4:**
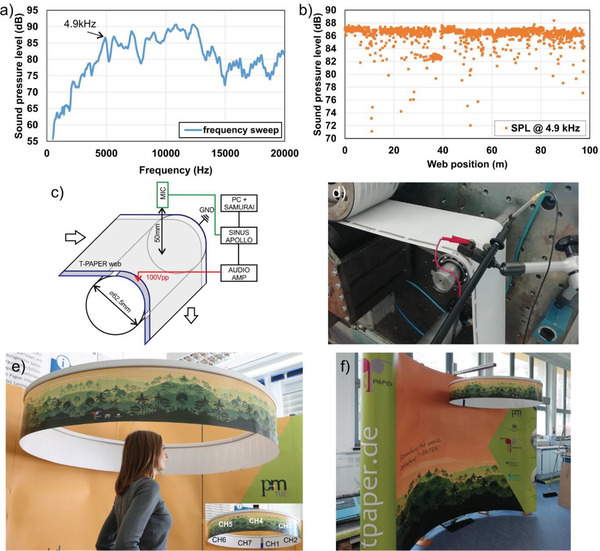
a) Frequency response of a single T‐PAPER cell (6 × 10 cm^2^). b) Acoustic inline measurement results of the peak SPL @ 4.9 kHz for a 100 m long T‐PAPER web. c) Schema of the acoustic inline measurement setup. d) Image of the acoustic inline measurement at the R2R printing press. e) 360° rain forest sound installation made of a 3.87 m long T‐PAPER web consisting of 56 cells, connected to 7 independent speaker segments/channels having 8 cells connected in parallel each (inset shows channel distribution). f) The 360° sound installation combined with a curved mobile booth.

It should be noted that the inline measured SPL of each individual cell for the 4.9 kHz signal showed a not absolutely flat behavior with a clear peak which is related to the bending of the cell while running around the guiding roller and the changing distance between the microphone and the moving center point of the cell (Figure S6, Supporting Information). A schema and an image of the acoustic inline measurement set‐up at the R2R printing press is displayed in Figure 4c,d. Additionally, a video of the inline acoustic measurement is available in the Supporting Information.

The high yield makes it possible to realize unique loudspeaker web installations of several meters length. We designed a circular installation consisting of 56 loudspeaker cells connected to seven individually driven loudspeaker modules consisting 8 speaker cells each, in total 3.87 m long (Figure 4e). The connections were made with R2R flexo printed silver lines fabricated in the same way like the contact pads of the piezoelectric cells. The web was connected into a ring shape and has a weight of only 141 g. As both surfaces of the loudspeaker paper remain white after all functional printing and post‐press steps they can be illustrated with individual graphics by means of conventional inkjet printing. In our case we chose a rainforest illustration to create a perfect 360° illusion of a rainforest scenery. Standing in the middle of the ring, one is surrounded by the sound of moving wild animals, chirping birds, and rainfall (video of the installation available as Supporting Information). Furthermore, the printed loudspeaker installation can be easily integrated to commercial mobile booths as shown in Figure 3f.

These results clearly indicate the high potential of our fully R2R manufactured paper‐embedded piezoelectric devices for novel audio applications.

In conclusion, paper‐embedded flexible and lightweight piezoelectric large‐area devices have been demonstrated including their highly efficient production process consisting of R2R methods only. While the functional layers were R2R printed, full device functionality was achieved by R2R lamination and R2R polarization. The novel T‐PAPER device set‐up guarantees high yield (≈93%) and excellent process stability (≈2–3% deviation) in combination with high remnant polarization (up to 78 mC m^−2^) of the laminated polymeric piezoelectric layer proven by different inline measurements. T‐PAPER webs could be used for unique 360° multi‐channel sound installations. On the one hand, the work paves the way for an economic and ecologic manufacturing of large‐area piezoelectric devices on basis of conventional paper. Besides the usage as speaker, the T‐PAPER concept can be used for much more applications in the field of flexible thin film sensors and actuators. On the other hand, it shows the sense and necessity for further optimization on the materials side, for example, for the development of piezoelectric materials/layers with faster switching mechanism and even higher electric breakdown field leading to a higher remnant polarization in shorter time. The mass printing of highly conductive layers made of polymers (or alternative electrode materials which allow polarization at high electric field) on conventional (untreated) paper seems to be of high importance for future research as well.

## Experimental Section

### R2R Printing

All printing steps were done at the R2R printing press LaborMAN 2 at the Institute for Print and Media Technology at TU Chemnitz.

At first, large‐area electrodes (6 × 11 cm^2^ each) were printed by means of rotary screen printing in a continuous pattern on 140 mm wide double‐sided pre‐coated paper with a grammage of 135 g m^−2^ and a thickness of 100 µm (maxigloss, IGEPA). The printing unit is an in‐house development to be compatible with the LaborMAN printing press design. As electrode material, a screen printing ink of the conductive polymer PEDOT:PSS was used as received (EL‐P 3155, AGFA). For a proper ink flow and better film forming, the formulation was stirred for 2 h prior to printing. Ink was continuously fed to the screen printing cylinder by a pressure controlled tank (Walther Pilot MDG1). More than 500 m were printed at a printing speed of 10 cm s^−1^. The PEDOT:PSS ink was dried in a hybrid way leading to a final layer thickness of ≈400 nm. For a very quick pre‐drying for 2 s, the paper web passed a NIR dryer (Adphos) directly after the printing unit. Additionally, the electrodes were annealed inline for ≈80 s in a conventional hot‐air dryer (140 °C). The stability of the process was controlled by inline sheet resistance measurements. For this, 4‐point measurements were done using a self‐made contact plate with four equidistant pins and a SMU in 4‐wire mode (2612, Keithley).

The piezoelectric layer was realized by rotary screen printing of P(VDF‐TrFE), (FC25, Piezotech Arkema) with a VDF:TrFE mol%‐ratio of 75:25. The powder was dissolved in a high‐boiling point solvent for long printing run stability. Depending on the target layer thickness (5–10 µm), the co‐polymer concentration of the ink was set between 15 and 25 wt%. Like for the PEDOT:PSS, P(VDF‐TrFE) ink was fed to the printing unit by the same pressure controlled tank. After printing, the web containing the wet co‐polymer layer run at a speed of 5 cm s^−1^ through the printing press for 80 s prior the drying process which was beneficial for a proper film forming and leveling to close pinholes typically visible directly after the printing unit. The drying process itself was done with the hot‐air inline dryer for ≈160 s at a temperature of 150 °C.

Finally, contact pads at one edge of the PEDOT:PSS electrodes were made of silver ink (Loctite ECI 1011 E&C, Henkel) by means of rotary flexo printing at a printing speed of 5 cm s^−1^, followed by an inline hot‐air drying procedure for ≈160 s at a temperature of 170 °C. The stability of the silver print was controlled by inline sheet resistance measurements. For this, 2‐point measurements were done using a self‐made contact plate with two pins and a SMU in Ohm‐meter mode (2612, Keithley).

The three aforementioned layers were realized in three subsequent more than 500 m long printing runs. Hence, after each printing trial the paper roll was rewound. Before the following lamination, the paper web was roll cut at one edge to reduce the web width to 125 mm which facilitates the later inline polarization easier. Additionally, the pre‐printed paper roll was split into two 260 m long rolls.

### R2R Lamination

R2R lamination of the two rolls was done at LaborMAN 1. Besides four printing units, the machine consisted of four winding units as well as a hot lamination unit. Thus, up to three (pre‐printed) webs could be combined to a final one.

To complete the T‐PAPER roll, the pre‐printed rolls were fed to two separate unwinding units. Both webs with the pre‐printed surfaces face to face and a parallel offset of 15 mm (to remain accessible contact pads) were brought into contact in the nip of the lamination unit consisting of a hard, temperature controlled cylinder with a diameter of 220 mm and a smaller roller working as impression cylinder with a soft rubber surface. The following process parameters were set for the web speed, the temperature of the lamination cylinder and the pressure of the impression roller, respectively: 3 cm s^−1^, 160–180 °C, 1 bar. The alignment of the webs was controlled by multiple web edge controllers (alignment across printing direction) and independent control of the web tension before and after lamination (alignment in printing direction). The stability of the lamination process (in combination with all printing steps) was controlled by inline capacitance measurements. The capacitance measurements on the running web were performed by using a self‐made opamp‐based oscillator circuit and a data acquisition system. The laminated webs were rewound once more.

### R2R Polarization

R2R inline polarization was done with the help of a self‐made fully automatic inline contact poling and measurement system integrated to a printing unit of the LaborMAN 2. In short, the system consists of two stages, one for detecting the position of the individual cells and measuring the capacitance of the cells and the second one for poling in an adapted way for each single cell. Contacting of the cells is done by a pair of contact pins, one contacting the silver pads of the web from the top, one from the bottom side for each stage. Each cell was polarized with ten full cycles of sinusoidal waves with a frequency of 2 Hz whereas the maximum peak voltage was set to 0.6, 0.9, 1.2, and 1.2/1.5 kV_p_ for the four executed polarization runs, respectively. The HV was provided by means of a high‐voltage amplifier (Model 5/80‐HS, Trek), and a standard HV resistor of 987 Ω was inserted between the sample and the grounding. The current flowing through the sample was determined by measuring the voltage across the HV resistor. The specific polarization cycles are explained in more detail in Figure S1, Supporting Information. The web speed was set to 1 cm s^−1^. Hysteresis measurements were taken simultaneously by using a self‐made Sawyer–Tower circuit and a DAQ hardware (PCIe 6321‐ National Instruments) for data acquisition. The current determined in this study is given by *I* = *I*_p_ + *I*_cap_ + *I*_con_, where *I*_p_, *I*_cap_, and *I*_con_ represent the current from dipole orientation, capacitive charging, and conduction of the sample, respectively. The polarization was determined by integrating the current over time. In the offline polarization scheme, a bipolar butterfly voltage waveform was applied in order to get rid of the contributions from capacitive charging and conduction process.^[^ ^23^, ^29^, ^30^ ^]^ In the inline polarization scheme, a sinusoidal voltage waveform was applied in order to save time, and the contributions of *I*_cap_ and *I*_con_ were removed through the method described in ref. ^[^ ^30^ ^]^. There was no big difference between hysteresis curves determined by both polarization schemes.

### R2R Acoustic Measurements

R2R acoustic measurements were conducted directly at the printing press LaborMAN 2. The polarized web was guided around a roller (diameter 62.5 mm) with an enlacement of ≈90°. A sinusoidal wave (4.9 kHz, 100 V_pp_) was continuously applied to the contact pads of the individual cells. The signal was generated by an acoustic analysis system (Apollo Box with Samurai software, SINUS Messtechnik) and an audio amplifier (PA‐1122, Monacor). The SPL was measured with a microphone (M301E, Microtech Gefell) placed 5 cm above the paper web surface connected to the Apollo Box. The web speed was set to 2 cm s^−1^. Additionally, the audio frequency response of single cells was measured with the same set‐up but in a static way without moving the web applying a linear frequency sweep from 500 to 20 000 Hz.

## Conflict of Interest

The authors declare no conflict of interest.

## Supporting information

Supporting Information

Supplemental Video 1

Supplemental Video 2
