# Supplementary material for: Paper‐Embedded Roll‐to‐Roll Mass Printed Piezoelectric Transducers
Source: Adv Mater. 2021 Jan 18;33(8):2006437. doi: 10.1002/adma.202006437 (PMC11468813; doi:10.1002/adma.202006437)
Supplement: Supplementary file 1 — Supporting Information [file ADMA-33-2006437-s002.pdf]

© 2021 Wiley-VCH GmbH

ADVANCED MATERIALS

Supporting Information

for *Adv. Mater.*, DOI: 10.1002/adma.202006437

Paper-Embedded Roll-to-Roll Mass Printed Piezoelectric Transducers

Georg C. Schmidt, Pramul M. Panicker, Xunlin Qiu, Aravindan J. Benjamin, Ricardo A. Quintana Soler, Issac Wils, and Arved C. Hübler*

Supporting Information

Paper-Embedded Roll-to-Roll Mass Printed Piezoelectric Transducers

Georg C. Schmidt, Pramul M. Panicker, Xunlin Qiu, Aravindan J. Benjamin, Ricardo A. Quintana Soler, Issac Wils, Arved C. Hübler*

Inline polarisation procedure

The R2R manufactured T-PAPER roll was inline polarised with a specific procedure to maximize remanent polarisation and yield. For each polarisation step, a HV signal with sinusoidal waveform and a frequency of 2 Hz was applied. The amplitude of the HV signal was increased within the first 5 cycles followed by 5 additional cycles of maximum amplitude. Thus, the polarisation takes 5 seconds per cell. As the contact pads have a length of 6 cm per cell, there is a time slot of 6 seconds at a web speed of 1 cm s^{-1}. Hence, the poling was triggered in a way, that each cell is in contact with the poling unit 0.5 s without HV signal followed by 5 s of poling followed by 0.5 s without HV signal. This procedure gives some headroom for misalignments between running web and polarisation unit (**Figure S1**).
To control the poling and to extract the remanent polarisation per cell, the current flowing through the cell was simultaneously extracted by measuring the voltage across the resistor, which is in series to the cells. The extraction of poling current and calculation of remanent polarisation were automated using MATLAB.

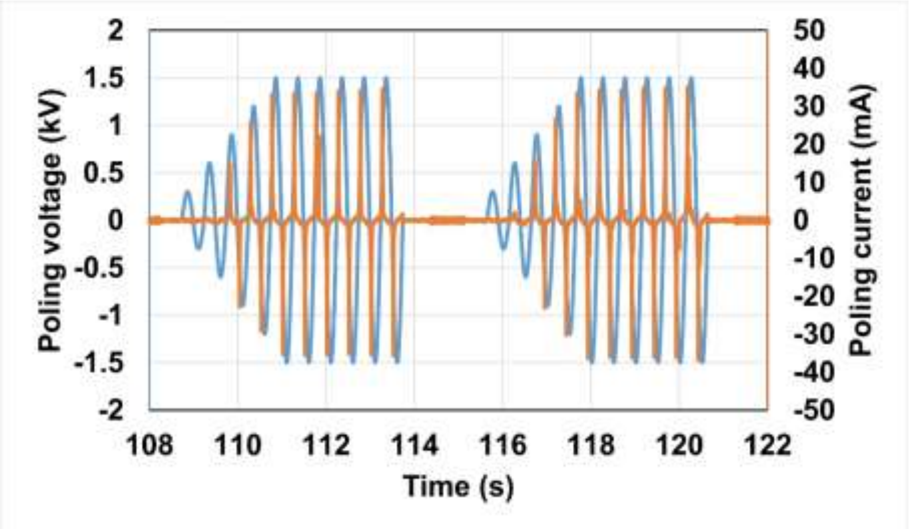

Figure S1. Inline poling procedure of two consecutive T-PAPER cells.

Hysteresis curves of inline and offline poling

The following graphs (**Figure S2**) show the hysteresis plots of an offline polarisation test (triangular waveform used) and the inline polarisation (sinusoidal waveform used) of R2R manufactured T-PAPER cells. Applying the same polarisation voltage results in very similar values for the remanent polarisation. Hysteresis loops for the inline polarisation were calculated from the last of ten voltage cycles (see Figure S1).
Additionally for the offline poling test, here the poling voltage before electrical breakdown was maxed out up to an electric field of ≈154 V μm^{-1}. This extremely high value for a large area piezo device of 60 cm^2 active area leads to a very high remanent polarisation of 78 mC m^{-2}.

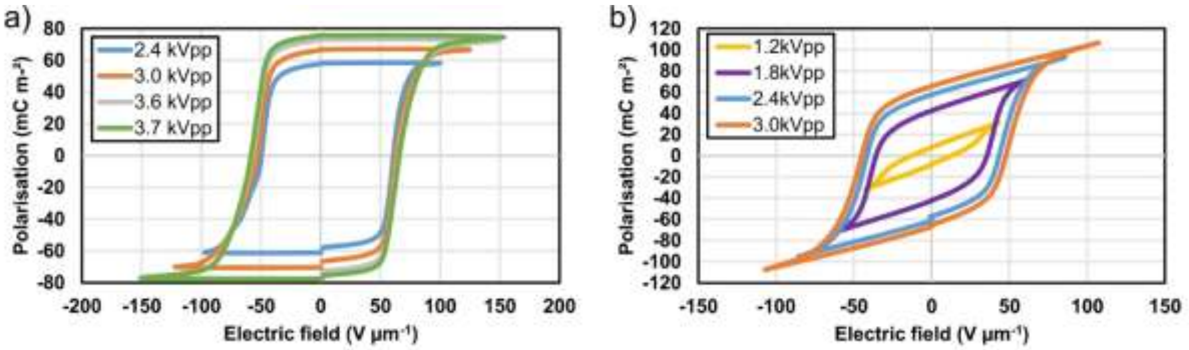

Figure S2. Hysteresis loops for a) the offline test polarisation and b) the inline poling of R2R manufactured T-PAPER cells.

Distribution of achieved remanent polarisation under variable inline polarisation voltage

The following graphs (**Figure S3-S5**) give a more detailed overview of the distribution of the achieved remanent polarisation depending on the amplitude of the poling voltage for the third and fourth inline poling run.

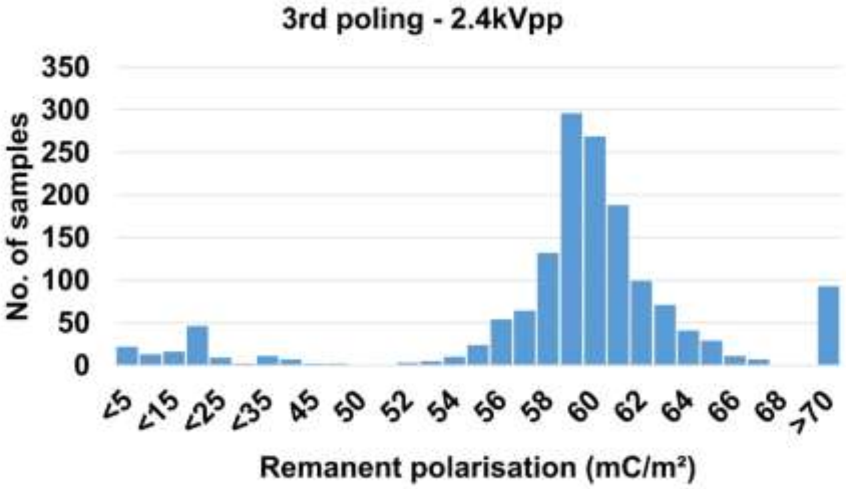

Figure S3. Distribution of the achieved remanent polarisation of T-PAPER cells for the third poling run at a maximum poling voltage of 2.4 kV_{pp}.

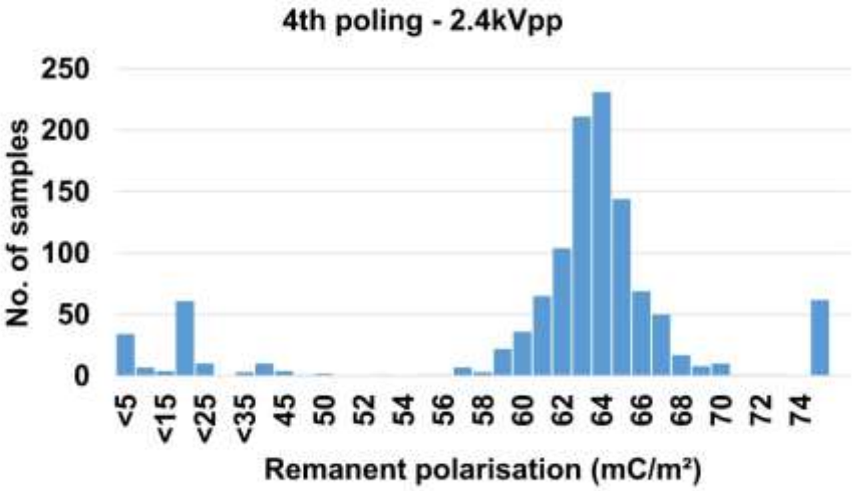

Figure S4. Distribution of the achieved remanent polarisation of T-PAPER cells for the fourth poling run at a maximum poling voltage of 2.4 kV_{pp}.

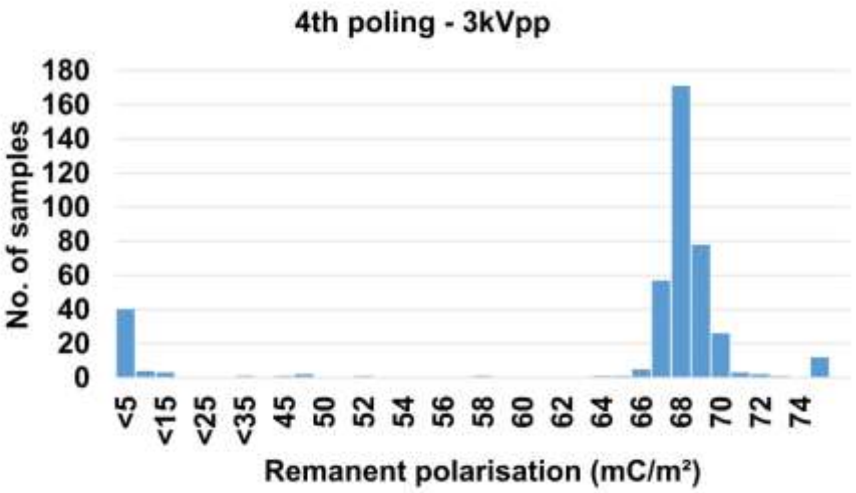

Figure S5. Distribution of the achieved remanent polarisation of T-PAPER cells for the fourth poling run at a maximum poling voltage of 3.0 kV_{pp}.

Acoustic inline measurements

All T-PAPER cells were acoustically characterised directly at the printing press. The measurements set-up is displayed in Figure 4c and Figure 4d. In the following, more information is given regarding the inline measured audio signal with respect to the position of each T-PAPER cell to the microphone and to the bending condition.
Figure S6 considers four different positions: (i) contact pins providing the 4.9 kHz, 100 V_{pp} signal are in the gap between two cells, i.e. there is no contact to the silver contact pads; (ii) contact pins just contact the beginning of the silver contact pads; (iii) contact pins are in the middle of the 60 mm long contact pads; (iv) contact pins reach the end of the contact pads.

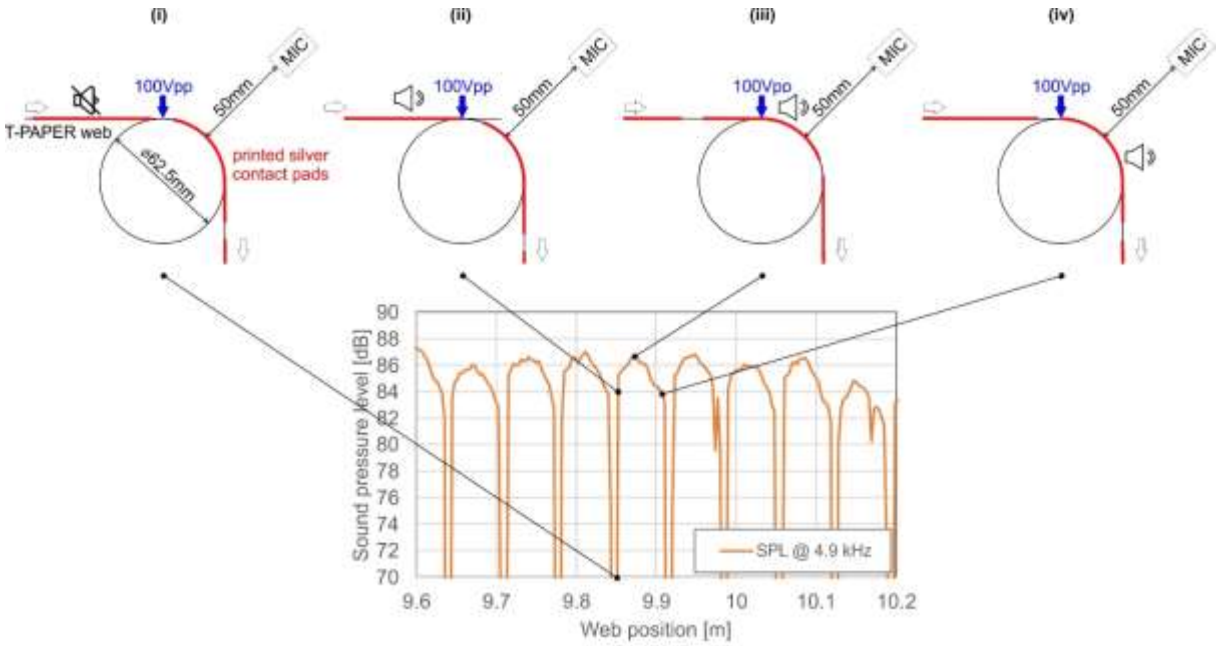

Figure S6. Geometry of inline acoustic measurement system with T-PAPER web and printed silver contact pads, guiding roller, microphone. The measured SPL is dependent on the web position. Four different web positions of one representative T-PAPER cell are drawn and linked to the inline measured SPL. The graph shows the SPL of 8 consecutive T-PAPER cells.
